# Food avoidance and restriction in adults: a cross-sectional pilot study comparing patients from an immunology clinic to a general practice

**DOI:** 10.1186/s40337-017-0160-4

**Published:** 2017-09-18

**Authors:** Michael Fitzgerald, Brad Frankum

**Affiliations:** Western Sydney University, Parramata, Australia

**Keywords:** Avoidance, Restriction, Food, Adult, Arfid, Intolerance, Allergy, Perceived

## Abstract

**Background:**

With the introduction of avoidant/restrictive food intake disorder (ARFID) in the Diagnostic and Statistical Manual – fifth edition, there is an increased need to understand the prevalence and pattern of food avoidance and restriction in adults. High rates of food allergy and intolerance in immunology clinic populations, and subsequent high rates of elimination diets, place these individuals at a greater risk of developing pathological eating behaviours. This descriptive cross sectional pilot study aims to provide preliminary data on the prevalence and nature of food avoidance and restriction in an adult population, and to explore the reasons for this behaviour.

**Method:**

A self-administered questionnaire was designed and distributed to adults presenting to an immunology clinic and a general practice over the course of 6 months to describe the prevalence and nature of avoidant and restrictive eating behaviours in this population. Pearson’s chi square test was used to examine the strength of a potential link to a formal diagnosis of avoidant restrictive food intake disorder in these patients.

**Results:**

A total of 102 completed questionnaires were used for data analysis. Food avoidance or restriction was detected in 81 respondents (79%), with rates not significantly higher in the immunology clinic group compared to the general practice group (*p* = .242). Food allergy and intolerance were the most common reasons for disturbed eating patterns. Life impact secondary to food avoidance and restriction was reported by 26% of respondents, with significantly higher rates observed in the immunology clinic cohort compared to the general practice (*p* = .011).

**Conclusions:**

Eating disturbances similar to those characteristic of ARFID are very common in adults. Food avoidance and restriction due to perceived food allergy and intolerance are significant reasons for such disordered eating patterns, particularly in an immunology clinic population. Further investigation is needed to determine if such eating behaviours are pathological and whether they qualify for a diagnosis of ARFID.

**Electronic supplementary material:**

The online version of this article (doi:10.1186/s40337-017-0160-4) contains supplementary material, which is available to authorized users.

## Plain English summary

Abnormal eating behaviours such as “fussy” or “picky” eating have traditionally been thought to exist specifically in childhood populations. However, recent developments in the diagnostic tools used by medical professionals to characterize eating disorders have recognised the existence of these eating behaviours in adults. This is unsurprising when one considers the growing acceptance of dietary requirements that involve the avoidance or restriction of certain foods in today’s society. Whilst food avoidance and restriction is often the product of medical or cultural motives, a developing concern is that a significant proportion of these behaviours may actually represent a newly described form of eating disorder known as Avoidant/Restrictive Food Intake Disorder. Accurate diagnosis of such disorders is vital to allow for appropriate treatment, rather than reinforcing the behaviour by accommodating or encouraging such restrictive diets. This is of particular importance for those populations of adults with higher rates of these diets, such as those with perceived or confirmed food allergy and intolerance. With the current lack of data exploring this phenomenon in the adult community, this study aims to measure how common these abnormal eating behaviours are across a few different adult populations, and explore the possible reasons for this.

## Background

The inclusion of Avoidant/Restrictive Food Intake Disorder (ARFID) in the Diagnostic and Statistical Manual of Mental Disorders (5th ed.; DSM-5; American Psychiatric Association, 2013), replacing and extending the previous ‘Feeding Disorder of Infancy or Early Childhood’, reflects recognition of the significant, growing number of people in the community who exhibit food avoidant and restrictive eating behaviours. According to DSM-5 criteria, the eating disturbance in ARFID may refer to, but is not limited to, an apparent lack of interest in eating or food, avoidance based on sensory characteristics of food, and/or concern about aversive consequences of eating [1].

At present, there is a relative lack of depth to the existing literature on patients with ARFID compared to other eating disorders, with the majority of research limited to retrospective reviews of previous studies. These papers, which have been limited by their restriction to highly-selected child and adolescent eating disorder clinic populations, have suggested an ARFID prevalence ranging from 5 to 22.5% [2–5]. Additionally, a recent screening study in Switzerland of primary school children found 3.2% of participants reported features of ARFID (such as food avoidance) upon self-rating questionnaire [6].

Unfortunately, there are presently no prevalence data or adequate case definitions of ARFID in adult populations available in published studies. Interestingly, a recent paper exploring disordered eating in adults noted a substantial proportion of individuals identified as being picky eaters (35.5%), with these people significantly more likely to exhibit food avoidance or refusal based on sensory characteristics of food [7]. Although a definitive diagnosis of ARFID requires fulfillment of additional criteria, the likelihood of a positive association with picky eating has been previously suggested with a positive history of the behaviour being reported by almost one-third (29%) of children and adolescents with ARFID and several longitudinal studies observing the positive predictive value of early-onset restrictive eating disturbances in elevated risks of future eating disorder development [3, 8–10]. It follows that ARFID may be assumed a disorder that affects adults as well as children and adolescents, despite the current lack of confirmatory data.

With eating disturbances motivated by concerns regarding aversive consequences of eating consistent with an ARFID diagnosis, it is necessary to consider the role of food allergy and intolerance in the condition [1]. Food allergy (FA) describes an adverse immunologic response to a specific food protein [11], the most common of which include milk, egg, peanut, tree nuts, shellfish, fish, wheat and soy [12]. While the most reliable diagnosis requires confirmation of a clinical allergic reaction with a food challenge [13, 14], in practice laboratory investigations such as skin prick testing and serum specific IgE levels are more commonly used to supplement the clinical picture. Food intolerance (FI) describes any non-immunologic-mediated adverse reaction to ingestion of a food and has thus far been poorly studied [11]. Significant variation exists with trigger substances, as with reaction types, however usually involves some degree of non-specific gastrointestinal upset, fatigue, malaise and migraines [15]. As with FA, FI is most reliably diagnosed with confirmation of adverse reaction following food challenge, however more commonly the diagnosis is based on a suggestive history combined with good clinical response to a trial elimination diet.

The accurate determination of FA prevalence is impeded by the lack of studies applying reliable diagnostic methodologies, such as double-blind placebo-controlled food challenges (DBPCFC), to large unselected populations [13]. The limited pool of existing studies that utilise oral food challenges suggest an actual prevalence of 1–3.5% across all ages [16, 17]. Comparatively, public perception of FA as measured in studies using self-reported questionnaires suggest a higher prevalence, with estimates ranging from 13.3 to 16% [14, 17]. This difference between actual and perceived food allergy (PFA) has also been illustrated in a meta-analysis of 51 studies containing both child and adult data. The review found PFA measured at 3–35% of the population compared to 1–10.8% for challenge-confirmed FA [18]. Rates of self-reported or perceived food intolerance (PFI) as measured in community based studies have also evidenced a similar trend towards overestimating the actual prevalence as determined by studies that utilise DBPCFC (~20% compared to 0.7–1.8%) [16, 19]. Unsurprisingly, a similar relationship is noted in studies comparing actual FA and FA together (2.4%) with perceived adverse food reactions (4.9–61.5%) [16, 20, 21].

As elimination diets form the foundation of treatment in FA and FI, the consequence of high rates of PFA and PFI are corresponding high rates of food avoidance and restriction, often self-induced and -enforced. More than 20% of adults and children alter their diets due to PFA [18]. In a study of children with atopic dermatitis treated with milk elimination diets, the actual prevalence of milk allergy (4%) was demonstrated to be significantly lower than the number of patients prescribed such diets (24.2%), confirming excessive application of strict dietary control [22]. Additionally, a survey of mothers regarding FA found 17% reported issues with adverse food reactions in their household, with nearly three quarters of altering their family’s eating habits as a result – despite the absence of professional diagnosis or recommendation [23]. These studies demonstrate that the simple perception of food allergic disease is sufficient to influence individual eating behaviours.

The nutritional hazards associated with rigorous adherence to elimination diets, particularly in children and independent of the verity of FA or FI, are well documented [24–27]. Interestingly, when prompted by an incorrectly perceived FA or FI, these behaviours also potentially satisfy a diagnostic criteria for ARFID. The question then arises – can the disparity between actual and perceived FA/FI prevalence and the subsequent high levels of food avoidance and restriction be, at least partially, better explained by this new diagnosis of ARFID? Whilst an association with ARFID itself has not yet been explored, there is precedent for suggesting that misperception of FA and FI may be the manifestation of psychopathology. A study by Pearson et al. [28] comparing the characteristics of patients at an allergy clinic with confirmed and unconfirmed allergy detected a high incidence of psychiatric disorder amoung patients with PFA that could not be confirmed with food challenge, while no psychological symptoms were reported in the group with confirmed FA. These findings were reproduced in another study that demonstrated a higher prevalence of psychiatric disorder in patients with PFA where a hypersensitivity reaction could not be confirmed by laboratory tests [29]. Adding further weight to the proposed link was that this group of patients were observed to be almost identical, in general characteristics and psychiatric symptomatology, with a comparative cohort of new psychiatric outpatient referrals. Since these earlier papers, a large cross-sectional and longitudinal study has also demonstrated the association between food allergies and symptoms of psychopathology, specifically including, but not limited to, eating disorders [30]. A similar association with psychopathology has also been established for FI with a study by Knibb [31] revealing significantly higher levels of psychiatric symptoms in patients with self-reported FI compared to individuals not reporting any adverse food reactions. Interestingly, some of these papers identified an increased prevalence of comorbid anxiety disorders in their patients – a finding mirrored by the recent papers examining the characteristics of patients with ARFID [3–5, 30, 31]. That both patients with ARFID and patients with PFA/PFI share high rates of psychiatric comorbidity strongly suggests the likelihood of non-mutual exclusivity in the two groups, and that the restrictive eating patterns of individuals with PFA/PFI may be better explained as undiagnosed eating disorder behaviour.

Achieving an accurate diagnosis in patients who exhibit disordered eating behaviours has significant clinical consequences. Primarily, excluding the presence of FA or FI in patients whose behaviour is motivated by a perceived need to avoid an adverse food reaction would help reduce the prevalence of unnecessary restrictive, elimination diets and the harmful physiological sequelae. Furthermore, confirming the diagnosis of an eating disorder, such as ARFID, facilitates more appropriate management. Several paediatric and adult case studies have illustrated the success of cognitive behavioural therapy including systematic desensitisation with in-vivo exposure, cognitive restructuring techniques, and psychoeducation in achieving improvements in nutritional adequacy, dietary expansion and self-management of food anxiety [32–34]. Further research into this relatively new disorder will only contribute to our understanding of how to best treat these patients.

The starting point for a diagnosis of ARFID necessarily rests with identifying self-imposed food avoidance and restriction, and any attempt to quantify the prevalence of ARFID in an adult population can only be informed by accurate data on such a disturbance in eating behaviours. Given the dearth of literature into such behaviours, this pilot study was designed to provide preliminary data on the prevalence and nature of food avoidance and restriction in an adult population, and explore the reasons for this behaviour. Additionally, the study aims to test the hypothesis from expert observation at the local immunology and allergy service in combination with suggestion in the current literature, of significantly higher rates of food avoidance and restriction in its clinic population compared to the wider, general community.

## Methods

A review of the available literature and current eating disorder questionnaires did not yield any established, validated tools to qualify the nature of, and reasons behind, food avoidant and restrictive behaviour. However, a newly developed and recently validated self-rating scale questionnaire sensitive to the diagnosis of ARFID and other eating disorders as per DSM-5 criteria, the Munich ED-Quest [35], was identified by the search. This tool, in combination with expert opinion and available literature on best questionnaire design [36, 37], was used to develop a self-administered questionnaire designed to gather demographic data, screen for food avoidance and restriction in adults, and allow for semi-qualitative analysis of the nature and reasons behind these behaviours (Additional file 1: Appendix 1).

The questionnaire was distributed across two sites – an immunology clinic in the Sydney Metropolitan Area and a general practice (GP) clinic in a regional NSW town – from July to December 2015 and July to September 2015 respectively. Patients aged 18 years and over were eligible for the study regardless of reason for clinic presentation. There were no exclusion criteria. Follow up with each site was performed at regular intervals throughout the collection stage. Review of results and initial feedback from collection sites early in the project identified some issues with readability of survey design and flow of question prompts. A second design with minor structural modifications was created to address these issues and distributed at the same sites in place of the original questionnaire (Additional file 1: Appendix 2).

An information sheet and consent form were provided to each participant prior to completion of the questionnaire. Signed consent forms were returned with the questionnaires but kept independent of their corresponding survey. Ethics approval for the project was provided by the Western Sydney University Human Research Ethics Committee as an amendment to the research protocol H9067.

Statistical analyses were performed using IBM SPSS for Windows (version 22.0; IBM Corp., Armonk, NY, USA). Prior to analysis, non-parametric assumptions of independence and expected frequencies were checked. Where possible, relevant variables with small sample size and greater than two categorical options were collapsed to meet the expected frequency assumption. Where assumptions were met, associations between specific demographic variables, avoidance behaviours and likelihood of ARFID were explored using Pearson’s chi-square test, while the Fisher’s Exact Test was utilised where expected frequency assumptions remained unsatisfied. *P* values less than 0.05 were considered statistically significant. Association strength was reported using the phi coefficient.

## Results

A total of 103 people completed the questionnaire during the study period. One survey was excluded from the final data analysis due to incomplete responses, leaving 102 completed surveys for data analysis. The number of people who declined to participate in the survey was not collected and hence a response rate could not be calculated.

### General demographics

The general practice population produced 42 respondents (41%) whilst the remaining 60 respondents (59%) were from the general immunology clinic population (Table 1). There were more than twice as many female as male respondents, with 70 females and 31 males comprising the study population. Almost one third of respondents were less than 30 years old (33%). Distribution was spread relatively evenly throughout the other age groups. The majority of respondents (55%) were from outside of the Sydney Metropolitan Area (SMA), with 45% of respondents living within Sydney.

**Table 1:** Demographics

Patient Demographics	*N* = 102 (%)
Cohort	
General Practice	42 (41)
Immunology Clinic	60 (59)
Sex^a^	
Female	70 (69)
Male	31 (31)
Age^b^	
18–29	32 (33)
30–39	14 (14)
40–49	16 (16)
50–59	17 (17)
60–69	12 (12)
70–79	7 (7)
Location^c^	
SMA	45 (45)
Outside SMA	54 (55)
Questionnaire Design	
Original	53 (52)
Modified	49 (48)

^a^ *N* = 101; ^b^ *N* = 98; ^c^ *N* = 99

### Prevalence of food avoidance or restriction

A total of 21 respondents (21%) reported no food avoidance or restriction, with 81 respondents (79%) indicating that they avoided or restricted certain foods in their diet. Of those who reported this eating behaviour disturbance, 38% were from the general practice population compared to 62% from the immunology clinic population, reflective of the overall proportion of these two sample groups in the study population.

Rates of food avoidance or restriction did not differ significantly between the two sample groups (*p* = .242, *X* ^*2*^ = 1.371, df = 1). In the general practice population, 74% (*n* = 31/42) of respondents reported food avoidance or restriction as compared to 83% (*n* = 50/60) in the immunology clinic population. Female respondents reported food avoidance and/or restriction more commonly than males (*n* = 58/70, 83% compared to *n* = 22/31, 71%), however the difference was not statistically significant (*p* = .174, *X* ^*2*^ = 1.844, df = 1). There was no significant association between age and rates of food avoidance and restriction (*p* = .333 FET), although people aged 30–39 reported a lower frequency of these behaviours compared to other age groups. Levels of food avoidance and restriction were comparable between respondents independent of their place of residence (*p* = .788, *X* ^*2*^ = .073, df = 1).

### Reasons for eating behaviour

The most common reasons provided for food avoidance and restriction are outlined in Table 2. Overall, food avoidance and restriction was most frequently attributed to concerns about aversive consequences of eating (*n* = 68/81, 84%) - *intolerance*, *allergy* and *makes me feel sick*. Avoidance based on the sensory characteristics of food (*n* = 24/81, 30%) such as taste, smell or texture, were reported relatively less commonly. Issues with functional dysphagia specifically, such as *stuck in throat* and *bad past experience*, were reported infrequently (*n* = 13/81, 16%). Intolerance was the most common reason for disturbed eating behaviour in both patient groups.

**Table 2:** Reason for Food Avoidance/Restriction^a^

	Total			General Practice			Immunology Clinic		
	*N* = 102	%	% (*n* = 81)^b^	*N* = 42	%	% (*n* = 31)^b^	*N* = 60	%	% (*n* = 50)^b^
Intolerance	44	43	54	12	29	39	32	53	64
Allergy	26	25	32	10	24	32	16	27	32
Makes me feel sick	26	25	32	8	19	26	18	30	36
Bad for me	22	22	27	6	14	19	16	27	32
Taste	18	18	22	7	17	23	11	18	22
Other	16	16	20	2	5	6	14	23	28
Smell	14	14	17	5	12	16	9	15	18
Bad past experience	11	12	14	4	10	13	7	12	14
Texture	9	9	11	2	5	6	7	12	14
Religion/Cultural	4	4	5	1	2	3	3	5	6
Stuck in throat	2	2	2	0	0	0	2	3	4
Colour	1	1	1	1	2	3	0	0	0
Appearance	1	1	1	0	0	0	1	2	2
Not available	0	0	0	0	0	0	0	0	0

^a^Multiple responses were allowed for this question; ^b^Percentage among respondents who reported food avoidance or restriction

Allergy, colour and taste were given more commonly as reasons for avoidance or restriction in the GP group than the immunology group.

Of the 102 total respondents, 25% (*n* = 26) and 43% (*n* = 44) reported avoiding or restricting certain foods as a result of allergy or intolerance, respectively. With respect to the 81 respondents who reported food avoidance or restriction, 32% reported allergy concerns as reason for their avoidance and 54% reported food intolerance as a factor (Table 2).

Rates of reported food avoidance or restriction due to allergy were the same across the GP and immunology clinic populations – 32% (*p* = .981, *X* ^*2*^ = .001, df = 1). However, rates of avoidance or restriction secondary to food intolerance differed significantly across the populations, with fewer GP clinic (39%) respondents reporting intolerance compared to immunology clinic (64%) respondents (*p* = .026, *X* ^*2*^ = 4.933, df = 1, phi = .247).

The most common reasons for food avoidance by sex were *intolerant* in females (*n* = 39/58, 67%) and *taste* in males (*n* = 9/22, 41%). Female and male respondents reported avoidance due to allergy at similar rates – 33% and 32% (*p* = .936, *X* ^*2*^ = .006, df = 1). Females were significantly more likely to give intolerance as a reason for food avoidance or restriction with 67% compared to 18% of males (*p* < .001, *X* ^*2*^ = 15.443, df = 1, phi = −.439).

Rates of reported avoidance and restriction due to allergy and intolerance were not associated with participant age (*p* = .764 and .254, FET respectively).

### Perceived food allergy

Avoidance due to allergy was reported in 26 respondents (Table 2). A clinical diagnosis had been made in 73% of these - by a specialist, GP or other health practitioner - while the remaining 27% had not yet been diagnosed by a clinician (Table 3). Of those who had received a clinical diagnosis of allergy, 11 (58%) reported positive skin prick tests. None of the respondents without clinically diagnosed allergy reported a positive skin prick test. Five respondents carried an adrenaline self-injecting device for their allergy. Only one respondent had an Australian Society of Clinical Immunology and Allergy (ASCIA) Anaphylaxis Action Plan.

**Table 3:** Charactersitics of Reported Food Allergy

Characteristics of Reported Allergy	*N* = 26 (%)
Clinical Diagnosis	
Undiagnosed allergy	7 (27)
Diagnosed allergy^a^	19 (73)
Specialist Immunologist/Allergist	12 (63)
General Practitioner	6 (32)
Other health practitioner	5 (26)
Positive Skin Prick Test^b^	
Yes	11 (44)
No	13 (52)
Unsure	1 (4)
Adrenaline Self-Injecting Device	
Yes	5 (19)
No	21 (81)
ASCIA Action Plan^b^	
Allergy	0 (0)
Anaphylaxis	1 (4)

^a^some reported diagnosis from more than one professional; ^b^N = 25

The presence of clinically diagnosed allergy explaining participant’s food avoidance and restriction did not differ significantly (*p* = .938, FET) between the general practice cohort (*n* = 7/10, 70%) and the immunology clinic (*n* = 12/16, 75%). Respondents from the immunology clinic were no more likely to have reported a positive skin prick test than those from the GP group (53% vs. 33%; *p* = .535, FET).

**Fig. 1:**
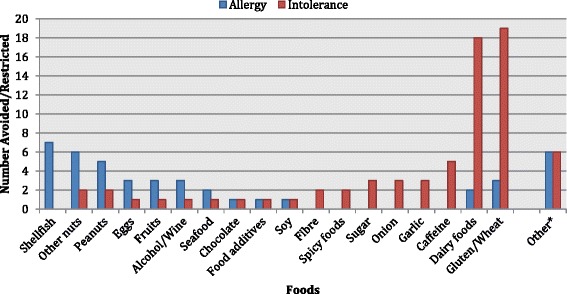
Most commonly reported foods avoided/restricted based on food allergy and intolerance. Multiple responses were allowed for this question. *Foods qualifying for ‘Other’ were only listed once

The foods most commonly avoided or restricted due to allergy are listed in Fig. 1. Shellfish, peanuts and other nuts were reported most frequently.

### Perceived food intolerance

Types of intolerance response to food are listed in Table 4. Bloating, abdominal pain and diarrhoea were the most frequently reported intolerance reactions. Excessive flatulence and nausea also featured prominently. Over one in ten people described allergic symptoms as components of their intolerance reaction. Some respondents who reported food avoidance and restriction secondary to the substance *makes me feel sick* and/or *other* also identified similar intolerance responses but are not included in Table 4.

**Table 4:** Food Intolerance Reactions

Types of Reported Intolerance Reactions	*N* = 44 (%)^a^
Bloating	32 (73)
Abdominal Pain	30 (68)
Diarrhoea	20 (45)
Excess Flatulence	15 (34)
Nausea	15 (34)
Headache/Migraine	6 (14)
Vomiting	5 (11)
Allergy type reactions (swelling of lips, hives, rashes/itch)	5 (11)
Constipation	3 (7)
Cramps	2 (5)
Gaseous indigestion	1 (2)
Sleep disturbances	1 (2)
Emotional mood fluctuations, irritability, difficulty concentrating	1 (2)

^a^Multiple responses were allowed for this question

The foods most commonly avoided or restricted due to intolerance are listed in Fig. 1. Gluten and dairy based products were reported most frequently.

### Exploration of link to ARFID: Impact of food avoidance and restriction

A total of 10% of all respondents felt their food intake was insufficient (*n* = 10/100). This percentage was marginally higher (11%) when considering the intake of those who reported food avoidance and restriction (Table 5). A lack of interest in eating was given as reason in 8 of these respondents - 7 of whom had also reported food avoidance and restriction. One respondent reported being unproductive at work as a result of their insufficient food intake. The majority of participants (*n* = 54/92, 59%) reported that their eating patterns, disordered or otherwise, did not impact their weight. Weight changes were more common in those with food avoidance and restriction (31%), with weight loss reported more frequently than weight gain (*n* = 17, 74% vs. *n* = 6, 26%). Changes in weight were not significantly associated with clinic cohort, sex or age (*p* = .645, .212 and .238, FET respectively). One respondent reported requiring a nasogastric (NG) tube to assist with feeding. The majority of respondents reported their food avoidance or restriction related to 3 or less foods or food groups (80%), while avoidance or restriction of 5 or more was only reported by 12% of respondents. The average number of foods avoided or restricted by respondents was 2.6.

**Table 5:** Significance of Eating Disturbance

Significance of Eating Disturbance	*N* = 81 (%)
Insufficient food intake	9 (11)
Lack of interest in eating	7 (78)
Unproductive at work	1 (11)
Weight disturbance^a^	23 (31)
Weight loss	17 (74)
Require NG feeds/nutritional supplementation^b^	1 (1)
Life Impact	21 (26)
Hard to go out to restaurants	14 (67)
Hard to attend social events	12 (57)
Hard to shop	5 (24)
Hard to cook	5 (24)

^a^ *n* = 75; ^b^ *n* = 80

Of the total respondents, 21% indicated that their life was affected by food avoidance or restriction (*n* = 21/101). The respondent who did not record an answer for this question had reported food avoidance due to shellfish allergy. With respect to those respondents who reported some degree of food avoidance or restriction, more than one in four felt impact on their life (26%). Difficulty in going to restaurants and social events were the most commonly indicated reasons for this difficulty (Table 5). Of the respondents with food avoidance and restriction, 38% reported at least one of either life impact or weight loss (*n* = 31/81), with seven participants reporting both.

Respondents from the immunology clinic cohort demonstrated significantly higher rates of life impact, or functional disturbance, secondary to their disordered eating behaviours compared to the GP cohort (*p* = .011, *X* ^*2*^ = 6.547, df = 1, phi = .286). Life impact was reported by 36% of those with food avoidance/restriction at the immunology clinic (*n* = 18/5) compared to only 10% from the GP sample (*n* = 3/30). Younger respondents, from the 18–29 years group, were also significantly more likely to experience life impact from their food avoidance or restriction compared to other age groups (*p* = .046 FET, phi = .385), with some age groups such as those aged 50–59 years demonstrating an opposite trend. No similar trend was observed with life impact across sex (*p* = .853, *X* ^*2*^ = .034, df = 1). Eating disturbance due to food allergy (*p* = .810, *X* ^*2*^ = .058, df = 1) and intolerance (*p* = .459, *X* ^*2*^ = .549, df = 1) was not associated with an increased rate of functional disturbance.

### Impact of questionnaire design

About half the respondents completed the original questionnaire, with the remaining half completing the modified questionnaire (Table 1). There was no significant difference between the rates of avoidance detected by the original questionnaire compared to the modified questionnaire (*p* = .154, *X* ^*2*^ = 2.037, df = 1). Reported levels of avoidance or restriction due to allergy (*p* = .489, *X* ^*2*^ = .479, df = 1) or intolerance (*p* = .273, *X* ^*2*^ = 1.204, df = 1) were also not significantly different between the two design groups.

## Discussion

To this author’s knowledge, this is the first study to collect data on the prevalence and nature of eating disturbances similar to those characteristic of the DSM-5 diagnosis ARFID in adults, with all previous studies into the new disorder and characteristic behaviours of food avoidance and restriction taking place in child and adolescent populations. Additionally, it is also the first study to examine the strength of a possible link between adult food avoidance and restriction and a diagnosis of ARFID, particularly from within an immunology clinic cohort.

Food avoidance and restriction was very common (79%) among adults. While adequate prevalence data for these specific patterns of disturbed eating is absent in the literature, this rate is considerably higher than what has been suggested. A similar study conducted in Switzerland with school children aged 8–13 years measured levels of disturbed eating behaviour characteristic of ARFID – food avoidance and restriction – of 3.2% (46/1444) [6]. The best indication of behavioural prevalence in an adult population is from a study exploring the characteristics of picky eating in adults, which found this behaviour was present in 35.5% of its participants [7]. Additionally, the most recent Australian census data in 2011–12 showed 17% of people aged 2 years or over reported avoiding foods due to allergy or intolerance [38]. The lower rates of food avoidance and restriction suggested by these sources, compared to the finding in this study, are likely a result of their participant recruitment from non-clinical environments. The use of care-seeking clinic populations in this study has the potential to introduce a selection bias and subsequently, overestimate the prevalence of disordered eating behaviour. Additionally, the observed association between ARFID and comorbid medical and/or psychiatric diagnoses in child and adolescent studies suggests that characteristic behaviours of restrictive eating and food avoidance could be more common in populations requiring general or specialist medical care [3–5]. The use of an unselected GP and immunology clinic patient cohort in this study limit the degree to which these findings may reflect prevalence rates in the broader population.

Rates of food avoidance and restriction were comparatively higher in the immunology clinic cohort (83%) than the GP clinic cohort (74%), albeit not significantly (*p* = .242). This is perhaps not surprising given the expected relatively higher load of patients in the immunology clinic cohort on elimination diets for food allergy or intolerance. Consistent with the DSM-5, no statistically significant difference in ARFID-like behaviours was detected between males and females (*p* = .174). This finding also mirrors the demographics of food avoidance and restriction in the literature. In the Kauer study [7], no difference in distribution of picky and non-picky eaters was observed with gender, and the Swiss paediatric study of ARFID-like eating behaviours also demonstrated an even split between genders [6]. However, the trend of slightly higher prevalence rates of food avoidance and restriction in females observed in this study has been previously documented. A study examining gender differences in the prevalence of eating disorder symptoms detected significantly higher levels of fasting, or excessive food restriction, reported by females [39]. The slight differences across sex, and also clinic populations, may be detected by a larger sample size, and further research in this area may demonstrate a more significant trend or pattern of food avoidance and restriction.

Food avoidance and restriction was most frequently attributed to concerns about aversive consequences of eating (84%), with food intolerance the most commonly reported reason in both clinic cohorts, and allergy and *makes me feel sick* also ranking highly (Table 2). Conversely, avoidance based on the sensory characteristics of food (30%) such as taste, smell or texture, were reported relatively less commonly. Interestingly, this pattern of eating disturbance contrasts with patterns in previous studies. Restrictive eating secondary to sensory properties of food was reported by 60.9% of participants in the Swiss paediatric study, with functional dysphagia occurring in only 15.2% [6]. Another paediatric study similarly estimated the prevalence of functional dysphagia far lower (9%) than restrictive eating based on sensory food properties (19%) [40]. The different pattern of food avoidance and restriction in this study is partially explained by the expected higher levels of intolerance and allergy in the immunology clinic population, even though participants were presenting for a variety of immunological conditions, not necessarily related to food. This is reflected in our finding of a significant, albeit weak, positive association between respondents from the specialist immunology clinic and reported food avoidance due to intolerance (phi = .247). Food allergy however was reported equally by respondents across both sample populations, and avoidance or restriction secondary to sensory characteristics of food like taste, smell and texture was also evenly distributed across both clinic groups. The difference in patterns of eating disturbance is probably more accurately accounted for by the limitations of the term ‘functional dysphagia’ as is used in the EDY-Q and ChEDE [6, 40]. Functional dysphagia refers to a conditioned negative response, and has been used to describe food avoidant conditions following or in anticipation of an aversive experience such as choking and feelings of food stuck in the throat [1, 41]. Using this definition, our study detected a similar low rate of functional dysphagia (*n* = 16%). However, the term does not adequately allow detection of other reasons for avoidance based on fear of negative consequences, significantly, such as adverse food reactions like perceived intolerance and allergy. As a result, food allergy and intolerance have been thus far under-investigated and under-reported, and probably represent a more significant motive for food avoidance and restriction in adults than has been reflected in the literature.

As mentioned above, the study identified a significantly greater proportion of avoidance due to food intolerance in the immunology clinic compared to the GP clinic. The finding has substantial clinical implications for practice and patient management. Specialist immunologists should be aware that their patient base are at a higher risk than the general population of adopting disordered eating behaviours that either are, or may become, pathological. Differentiating clinically between actual intolerance, which requires monitoring and management for subsequent dietary issues, and reported food intolerance that may be a manifestation of an eating disorder is essential. Pathological food avoidance may require counselling regarding safe foods or even referral to an eating disorder specialist for assessment and appropriate treatment such as psychotherapy. In all likelihood, the strength of the positive association between respondents from the immunology clinic and avoidance due to intolerance could have been greater if the reason for food avoidance/restriction “*makes me feel sick*” was not considered separately in this study. Combining these reasons in future studies may reveal a greater statistical significance and stronger association between the two groups.

The high levels of food avoidance and restriction in adults detected by this study, and specifically the comparatively higher rates in the general immunology clinic cohort, suggest that disturbed eating behaviours and furthermore eating disorders such as ARFID may be a significant issue in this population. However, one retrospective study of patients presenting to a paediatric gastroenterology clinic identified only 1.5% of cases (*n* = 33/2231) that met the complete diagnostic criteria for ARFID, demonstrating that even within a treatment-seeking cohort where ARFID-like eating difficulties are common and expected, the disorder is not over-inclusive [42]. In order to determine the likelihood of making a diagnosis in these patients, further examination of the diagnostic criteria for ARFID is required.

The primary diagnostic feature of ARFID involves avoidance or restriction of food intake manifested by clinically significant failure to meet nutritional requirements [1]. This failure may be indicated by weight loss, nutritional deficiency, dependence on enteral feeding or supplements, or by a marked interference with psychosocial function. Of our respondents with food avoidance and restrictions, almost 40% reported either a reduction in weight or some degree of functional impairment, with the latter response significantly more common among younger respondents and those from the immunology clinic. Dependence on enteral feeding or nutritional supplementation was only reported by one patient, however this was exclusively during the course of a separate illness, not as a result of individually driven food restriction. While complete satisfaction of this criterion requires assessment of the severity of psychosocial interference and the degree of the weight loss, which was beyond the scope of this study, the finding suggests possible eating disorders in these populations. Furthermore, with regard to quantifying the degree and extent of food avoidance and restriction as indicated by the number of foods and food groups avoided by this population, this study demonstrated a majority of people avoiding only a few foods or food groups (80% for 3 or less), compared to more comprehensive avoidance (12% for 5 or more). Although not a specific diagnostic criteria, it is highly likely that those individuals with more widespread food avoidance and restriction are at higher risk of satisfying the other criteria for a diagnosis of an eating disorder such as ARFID. With consideration of the proportion of respondents with ARFID-like eating disturbances who felt their food intake was insufficient (11%), the results allow for a closer comparison to early ARFID prevalence data from paediatric populations, which estimate rates of 5–22.5% [2–5]. Interestingly, the highest paediatric prevalence, detected by Nicely [3], was attributed to the study’s use of the proposed DSM-5 criteria to make diagnoses, which, similar to the survey tool used in this study, did not include the important body image distortion exclusion criteria for AN and BN. Although the findings of this study do not provide a complete measure of the prevalence of ARFID in adults, the high rates of clinical consequence (functional impairment, weight loss) from participant’s food avoidance and restriction in our population compared to the relatively lower prevalence estimated in the studies of more selected, specialist paediatric eating disorder clinics, suggest that ARFID may be more common in adults, or at least, significant. Further investigation directed at qualifying and quantifying rates of ARFID in adults, and particularly in immunology clinic patients and young adults, is indicated to gain a greater understanding of the disorder and its patterns.

The link from food avoidance and restriction to a formal diagnosis of ARFID also requires confirmation that the eating disturbance cannot be better explained by a concurrent medical condition [1]. In the context of this study, this process may involve identifying cases of perceived, but not actual, food allergy and intolerance, or assessing the degree to which food avoidance and restriction in the presence of genuine medical issues extends into excessive, pathological behaviour.

Food allergy was reported in a quarter of study participants, with a marginally higher rate in the immunology clinic population compared to the GP cohort. Food intolerance was more common, present in 43% of all participants, with a significantly greater proportion of immunology clinic patients reporting it as a concern compared to GP patients. It is difficult to confidently and accurately confirm actual adverse food reactions with a self-administered questionnaire, as was used in this survey, given the limited information about the clinical response that can be collected. Subsequently, there was a mixed indication of the verity of allergy and intolerance reactions reported. The self-reported rates detected in this study are consistent with the findings of similar previous studies that document a wide range of PFA and PFI prevalence as high as 3–35% [14, 17, 18] and ~20% respectively [19]. It is worth noting that much of the current literature in this area is difficult to align to this paper, and to each other, on account of varying quality and strengths, inconsistent use of terminology (eg: food allergy, food intolerance, adverse food reactions, food hypersensitivity, non-allergic food hypersensitivity), mixed child and adult populations, the consideration of allergy and intolerance as one entity, and the probable underestimation of true food intolerance rates. However, it is reasonable to conclude that the disparity between the high self-reported rates of food allergy and intolerance, and the lower DBPCFC confirmed rates (1–10.8% [16–18] and 0.7–1.8% [16, 19] respectively) probably suggest that a similar significant proportion of perceived, but not actual, food allergy and intolerance was detected in this study. This hypothesis is further supported by the low frequency of reported allergy that had been confirmed with positive skin prick testing (44%) in this study, and the substantial proportion of patients who reported food allergy in the absence of any professional diagnosis (27%). Although difficult to quantify, respondents within these groups in particular are those who, upon further examination, may qualify for a diagnosis of ARFID.

Importantly, even in cases where allergic disease and food intolerance is genuine, comorbidity with an eating disorder is neither impossible, or even unusual, with medically-endorsed dietary restrictions often progressing to become pathological eating behaviours [1, 43]. This is of particular significance given the consistency between the most commonly listed foods causing both allergy and intolerance in this study (Fig. 1), and the results of larger community data samples [12, 38, 44, 45]. Additionally, whilst specific allergy symptoms were not explored by the survey tool used in this study, the types of symptoms provided by those respondents reporting food intolerance (Table 4) were also consistent with expected reactions [15, 45]. Even when genuine, medically-endorsed food restrictions are commonly accompanied by types and degrees of disturbed eating independent of the condition that extend into the pathological behaviours necessary for a formal diagnosis of ARFID [1, 43]. Kauer’s study demonstrated this phenomenon, with individuals who listed medical restriction as the reason for their food refusal more likely to identify as picky eaters [7]. Definitive differentiation between actual allergy and intolerance, and the pathological food avoidance and restriction that manifests as perceived food reactions and self-imposed restrictive diets, requires more detailed assessment from clinicians in combination with objective investigations such as skin prick testing, food specific IgE and, where appropriate, food challenge.

The importance of differentiating between food allergy and intolerance that qualifies for a diagnosis of ARFID compared to that which doesn’t can also be applied to food avoidance based on sensory characteristics. With regard to the findings in this study, the 11% of respondents reporting avoidance due to food texture is likely to be a more significant and stronger indicator of possible ARFID diagnosis than avoidance due to taste (22%). Existing literature examining the characteristics of paediatric patients with ARFID have demonstrated a higher degree of food avoidance secondary to rejection of texture compared to those with other eating disorders [3]. More generally, food sensitivity related to texture is a common feature of other child mental disorders. In a study of parents of children with autism spectrum disorder, almost 70% felt that their child’s food selectivity was impacted by food texture – the most frequently reported sensory factor [46]. Although it is yet to be established whether texture sensitivity is limited to childhood populations or remain an important characteristic of adult food avoidance, it is possible that texture-based food avoidance may represent a non-specific indicator of psychiatric and eating disorder diagnoses. With Kauer’s study showing self-identified adult picky eaters as generally more likely than non-picky eaters to endorse food rejection based on texture, further investigation into the significance of texture-based food avoidance and ARFID in adults is indicated [7].

The final criteria required for a complete diagnosis of ARFID necessitates that the food avoidance or restriction be not better explained by a lack of available food or by an associated culturally sanctioned practice [1]. Rates of cultural or religious reasons for food avoidance comprised only 5% for people who indicated some degree of disturbed eating in this study, and unsurprisingly given the location of the study, no participants avoided or restricted foods due to lack of availability suggesting reasonable satisfaction of this diagnostic component in our population. As discussed above, confirmation that the pattern of disturbed eating did not occur exclusively during the course of AN or BN is a crucial exclusion criteria for ARFID [1]. As with the study by Nicely [3], this particular diagnostic feature was not adequately explored or excluded in our study and therefore limits the ability of our findings to reflect actual ARFID prevalence rates in adults. Subsequent research into ARFID in adults would be assisted by assessment for body image perception and/or disturbance. The EDY-Q, validated in the Swiss paediatric study on eating behaviours characteristic of ARFID, as well as the Munich-ED questionnaire for the diagnosis of eating disorders under the DSM-5, both include screening questions for AN and BN and would be appropriate starting points [6, 35].

The major limitation of this study is the unknown and potentially significant degree to which bias in the sample population affects the generaliseability and external validity of its findings. The use of a convenience sample of health treatment-seeking participants recruited from the Immunology Clinic and GP Practice may have inaccurately inflated the primary findings of food avoidance and restriction prevalence in adults. Furthermore, it is very possible that patients with a higher interest in food-related issues were over-represented in this study. However, as the time limitations placed on data collection and clinic reception staff in questionnaire distribution did not allow a response rate to be calculated, the degree to which this may have biased the results is difficult to quantify. Given the small final population examined, the conclusions of this study are likely to be heavily based on the participants who comprised the sample group and as such, free application of the findings to broader, general populations should be avoided until validation and replication of study methodology and results. The use of an unvalidated survey tool in the absence of an appropriate questionnaire to assess food avoidance and restriction in adults presented some issues with the design requiring minor modifications during the course of data collection. Although the two formats did not demonstrate any significant difference between levels of food avoidance and restriction detected (*p* = .154), the changes may have affected some of the smaller sub-group analyses. The primary difference made was with regard to placement of question cues, with the original survey resulting in participants who had indicated no food avoidance or restriction being asked to respond to questions not relevant to them including weight changes and presence of intolerance symptoms. To avoid any influence over findings, responses to these questions in the initial survey tool were interpreted in the context of whether or not patients had indicated they avoided certain foods. Finally, as a pilot study, the questionnaire was clear but only modest in its intent to diagnose ARFID. Future research should aim to determine accurate prevalence data on ARFID in adults using a larger, randomly selected sample to better reflect the general population.

## Conclusions

Food avoidance and restriction is very common in adults and the nature and reasons for these behaviours have thus far been poorly examined. The fear of aversive consequences, especially perceived food intolerance and allergy, are significant reasons for this disordered eating. These motivations are particularly significant within certain populations, specifically patients attending a general immunology clinic and females. Differentiating between actual food allergy and intolerance is particularly vital within these groups, as there is potential for progression to pathological levels of disordered eating. A proportion of adults with food avoidance and restriction suffer from the effects of insufficient dietary intake. This can be apparent clinically through weight loss or varying degrees of impairment in social functioning. Younger adults, and those attending an immunology clinic, may be more likely to report such impairment, and thus are the demographics that may require further assessment for a formal diagnosis and treatment of an eating disorder like ARFID. Further research into food avoidance and restriction in adults and the relationships between food intolerance, immunology clinic patients and pathological eating behaviours would be useful contributions to our developing understanding of ARFID.

## Additional file

Additional file 1: Appendix 1. Original questionnaire design. Appendix 2. Modified questionnaire design. (DOCX 390 kb)

## Abbreviations

AN: anorexia nervosa
ARFID: Avoidant/Restrictive Food Intake Disorder
ASCIA: Australasian Society of Clinical Immunology and Allergy
BN: bulimia nervosa
ChEDE: Child Eating Disorder Examination
DBPCFC: double blind placebo-controlled food challenge
DSM: Diagnostic and Statistical Manual
EDY-Q: Eating Disturbances in Youth – Questionnaire
FA: food allergy
FI: food intolerance
GP: general practice
NG: nasogastric
PFA: perceived food allergy
PFI: perceived food intolerance
SMA: Sydney Metropolitan Area
