# Supplementary material for: Food avoidance and restriction in adults: a cross-sectional pilot study comparing patients from an immunology clinic to a general practice
Source: J Eat Disord. 2017 Sep 18;5:30. doi: 10.1186/s40337-017-0160-4 (PMC5603184; doi:10.1186/s40337-017-0160-4)

Additional file

Additional file 1: **Appendix 1** Original questionnaire design


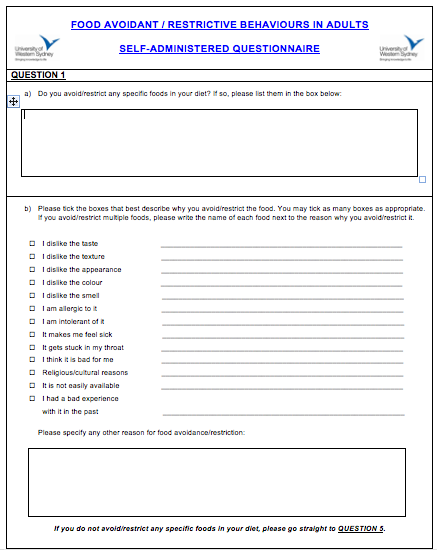


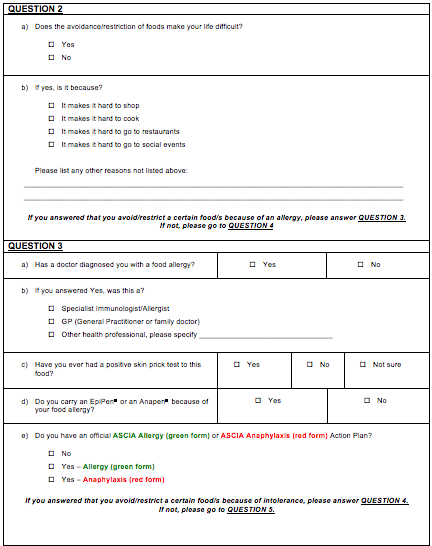


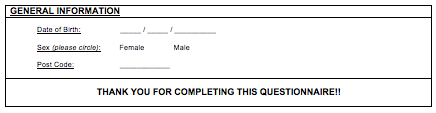

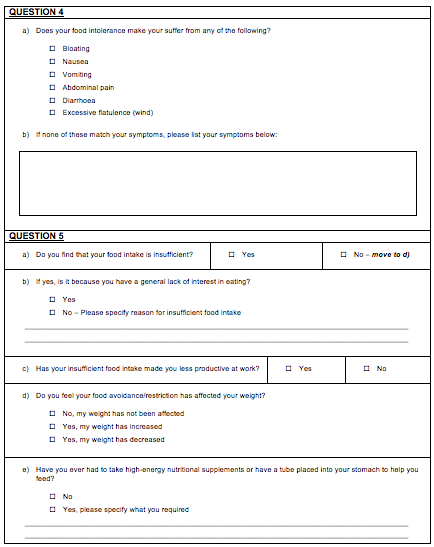


Additional file 1: **Appendix 2** Modified questionnaire design


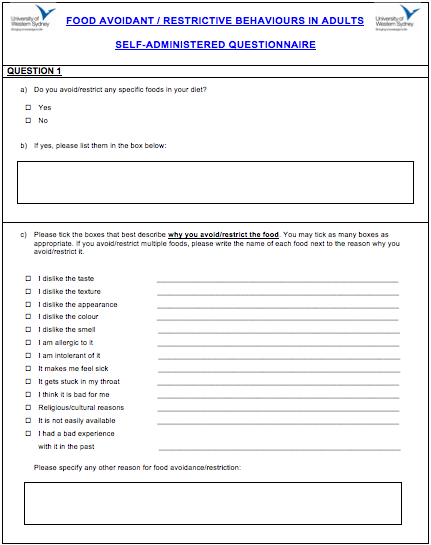


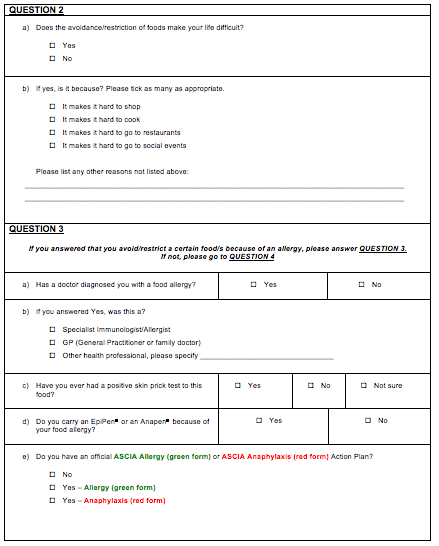


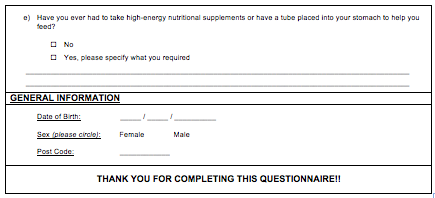

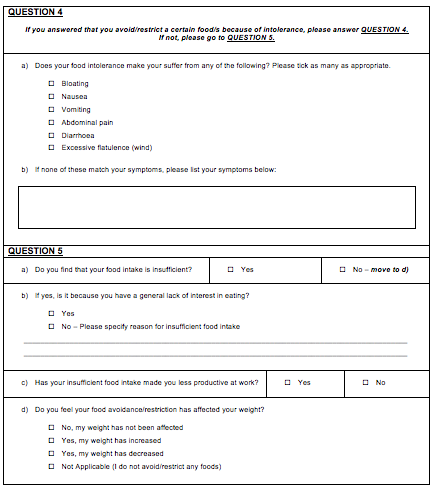

Supplement: Additional file 1: — Appendix 1. Original questionnaire design. Appendix 2. Modified questionnaire design. (DOCX 390 kb) [file 40337_2017_160_MOESM1_ESM.docx]
